# Endoplasmic reticulum−mitochondria coupling increases during doxycycline-induced mitochondrial stress in HeLa cells

**DOI:** 10.1038/s41419-021-03945-9

**Published:** 2021-06-28

**Authors:** Camila Lopez-Crisosto, Alexis Díaz-Vegas, Pablo F. Castro, Beverly A. Rothermel, Roberto Bravo-Sagua, Sergio Lavandero

**Affiliations:** 1 Advanced Center for Chronic Diseases (ACCDiS), Facultad de Ciencias Químicas y Farmaceuticas & Facultad de Medicina, Universidad de Chile, Santiago, Chile; 2 Advanced Center for Chronic Diseases (ACCDiS), Facultad de Medicina, Pontificia Universidad Católica de Chile, Santiago, Chile; 3 Charles Perkins Centre, School of Life and Environmental Sciences, The University of Sydney, Camperdown, 2050 Sydney, NSW Australia; 4 Corporacion Centro de Estudios Científicos de las Enfermedades Cronicas (CECEC), Santiago, 7680201 Chile; 5 Department of Internal Medicine (Cardiology Division), University of Texas Southwestern Medical Center, Dallas, TX USA; 6 Instituto de Nutrición y Tecnología de los Alimentos (INTA), Universidad de Chile, Santiago, 7830490 Chile; 7 Chilean State Universities Network on Aging, Universidad de Chile, Santiago, Chile

**Keywords:** Energy metabolism, Mitochondrial proteins

## Abstract

Subcellular organelles communicate with each other to regulate function and coordinate responses to changing cellular conditions. The physical-functional coupling of the endoplasmic reticulum (ER) with mitochondria allows for the direct transfer of Ca^2+^ between organelles and is an important avenue for rapidly increasing mitochondrial metabolic activity. As such, increasing ER−mitochondrial coupling can boost the generation of ATP that is needed to restore homeostasis in the face of cellular stress. The mitochondrial unfolded protein response (mtUPR) is activated by the accumulation of unfolded proteins in mitochondria. Retrograde signaling from mitochondria to the nucleus promotes mtUPR transcriptional responses aimed at restoring protein homeostasis. It is currently unknown whether the changes in mitochondrial−ER coupling also play a role during mtUPR stress. We hypothesized that mitochondrial stress favors an expansion of functional contacts between mitochondria and ER, thereby increasing mitochondrial metabolism as part of a protective response. Hela cells were treated with doxycycline, an antibiotic that inhibits the translation of mitochondrial-encoded proteins to create protein disequilibrium. Treatment with doxycycline decreased the abundance of mitochondrial encoded proteins while increasing expression of CHOP, C/EBPβ, ClpP, and mtHsp60, markers of the mtUPR. There was no change in either mitophagic activity or cell viability. Furthermore, ER UPR was not activated, suggesting focused activation of the mtUPR. Within 2 h of doxycycline treatment, there was a significant increase in physical contacts between mitochondria and ER that was distributed throughout the cell, along with an increase in the kinetics of mitochondrial Ca^2+^ uptake. This was followed by the rise in the rate of oxygen consumption at 4 h, indicating a boost in mitochondrial metabolic activity. In conclusion, an early phase of the response to doxycycline-induced mitochondrial stress is an increase in mitochondrial−ER coupling that potentiates mitochondrial metabolic activity as a means to support subsequent steps in the mtUPR pathway and sustain cellular adaptation.

## Introduction

Eukaryotic cells contain membrane-bound organelles that help to physically separate various cellular functions. Although these organelles were initially thought to be independent entities, delineated and defined by their membranes, the concept of communication between organelles is now widely accepted as a fundamental part of their operation [1–3]. Communication between organelles is a dynamic process in time and space that can be mediated by signaling molecules that are transferred from one organelle to another. The efficacy of communication can be impacted by changes in the physical distance between two organelles without either loss of individuality or fusing of membranes [1, 3]. Physical contacts between organelles are generally mediated by protein complexes that regulate the interactions and transfer of components between organelles [1, 3–6]. In previous reports from our group, we have shown how organelles communicate with each other and how this communication participates in various pathophysiological processes [7–13].

Interestingly, we showed that the early adaptive response to endoplasmic reticulum (ER) stress involves an increase in ER−mitochondria contacts, enhancing Ca^2+^ transfer from ER to mitochondria, and increasing mitochondrial metabolism [7]. This response is not limited to ER stress since we have observed similar results after treating cells with rapamycin, as a general metabolic stress [8]. Moreover, alterations in the communication between ER and mitochondria have been reported in numerous pathologies [14–19].

Mitochondria are semi-autonomous organelles that fulfill several functions, from cellular metabolism to regulating signaling and cell death [20]. Most mitochondrial proteins are encoded in nuclear DNA and must be imported from the cytosol. However, since mitochondria have their own DNA, they also code for a few proteins, which form part of the electron transport chain (ETC) complexes [21]. These proteins must be transcribed and translated in a coordinated manner with their nuclear counterparts and must be assembled correctly [20, 21]. Furthermore, due to the presence of the ETC and the highly oxidative environment of the mitochondrial matrix, mitochondria are especially susceptible to alterations in protein homeostasis [20, 22–24]. For this reason, mitochondria, as well as the rest of the cell, have evolved a variety of protein quality control mechanisms [25–27]. When there are alterations in protein homeostasis, adaptive genetic responses known as unfolded protein responses (UPR) are triggered, which vary depending on the place of origin of the stress [25]. In mitochondria, the existence of the so-called mitochondrial UPR (mtUPR) has been described in recent decades [22, 28–30]. Stressors that can trigger mtUPR include depletion of mitochondrial DNA, mutations in genes encoding mitochondrial proteases or components of the ETC, compounds that increase ROS production, and disruption of mitochondrial translation by interfering RNA or antibiotics such as doxycycline [20, 22, 30–33]. The mtUPR, a form of communication between mitochondria and nucleus (also known as retrograde, or mitonuclear communication), is thought to consist primarily of pathways that increase in expression of genes that help relieve stress, such as mitochondrial chaperones and proteases [20, 24, 30, 34]. Although many aspects related to the fine regulation and signaling of this response are still unknown, especially in mammals, the mtUPR has been given greater attention in recent years as it is proposed to play a fundamental role in various diseases, including infections, neurodegeneration, cancer, cardiovascular disease, metabolic disorders, and even the aging process [20, 22, 35–41].

Despite increased interest in the mtUPR, many aspects of the mechanisms of adaptation to mitochondrial stress at the molecular and intracellular level are still unknown. Mitonuclear communication has been widely described to trigger the transcriptional response comprising the mtUPR [20, 30, 42–44]; however, whether the changes in the ER−mitochondrial communication are a component of the adaptive response to mitochondrial stress remains unexplored. Here, we use HeLa cells to evaluate if doxycycline, an antibiotic known to induce mtUPR, can cause changes in the coupling between ER and mitochondria.

## Results

### Doxycycline treatment rapidly increases the physical coupling of mitochondria with ER

**Fig. 1:**
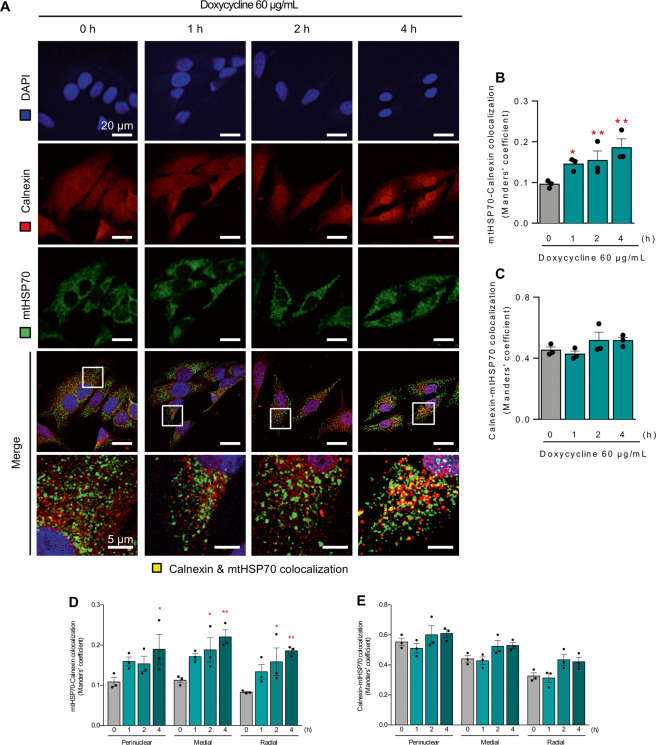
Doxycycline increases ER-mitochondria colocalization in a global fashion. HeLa cells were treated with 60 µg/mL doxycycline for the indicated times. **A** The ER was stained with anti-calnexin antibody (shown in red), mitochondria were stained with anti-mtHsp70 antibody (shown in green), and the nucleus was stained with DAPI (shown in blue) and then imaged using confocal microscopy. Colocalization is shown in yellow in the merged images. Scale bars: 20 and 5 µm (magnification). **B** Mitochondria-to-ER colocalization was quantified as Manders’ coefficients of images obtained in panel (**A**). **C** ER-to-mitochondria colocalization was quantified as Manders’ coefficients of images obtained in panel (**A**). **D**, **E** Quantification of the mitochondria-to-ER and ER-to-mitochondria Manders’ coefficients in the predefined subcellular regions (*n* = 3). Results are shown as mean ± s.e.m. *P < 0.05 and **P < 0.01 versus control condition.

Previously, we demonstrated that an early cellular response to stress in the ER or cytoplasm (treatment with tunicamycin or rapamycin, respectively) increased communication between ER and mitochondria [7–9]. Here, we sought to determine whether stress signaling originating in mitochondria would have a similar effect on the ER−mitochondrial interface. We chose to use doxycycline, an antibiotic of the tetracycline family that inhibits mitochondrial translation, thereby causing an imbalance in the mitochondrial proteome between the levels of mitochondrial and nuclear genome, known as mitonuclear protein imbalance [22, 33, 45]. As mtDNA encodes for subunits within four of the five mitochondrial ETC complexes, reducing the rate of translation of mtDNA encoded proteins increases the potential for excess nuclear-encoded proteins to form non-functional aggregates. We have previously demonstrated that under conditions of mild ER stress, there is an early adaptative response that involves an increase in contacts between ER and mitochondria [7–9]. To evaluate whether a similar increase in contacts between ER and mitochondria occurs early on in response to mitochondrial stress, we assessed the colocalization of mitochondria and ER by indirect immunofluorescence, using antibodies directed against mitochondrial Hsp70 and calnexin, an ER membrane marker. Our results show a time-dependent increase in the mitochondria-to-ER Manders’ coefficient (Fig. 1A, B). However, the ER-to-mitochondria Manders’ coefficient remains unaltered (Fig. 1C), suggesting that it is mitochondria that approach the ER and not vice versa. Interestingly, in contrast to what was previously described for tunicamycin-induced ER stress, where new ER−mitochondria contacts appeared almost exclusively in the perinuclear zone [7, 8], doxycycline increased ER−mitochondria contacts globally throughout the cell (Fig. 1D, E).

As a complementary approach, we used a split green fluorescent protein reporter (split-FP) to track changes in the physical proximity of these organelles using fluorescence microscopy. This tool, generously provided by Dr. Gyorgy Szabadkai (UCL, London), works on the principle that when the GFP protein is cut in two (GFP_β1–10_ and GFP_β11_), neither fragment is fluorescent on its own. However, fluorescence is restored when the two pieces come in close enough to one another. One portion of the split GFP was targeted to the ER (ER GFP_β11_) using either a short (~10 nm) or a long (~50 nm) linker. The other portion of the split GFP was targeted to the outer mitochondrial membrane (OMM GFP_β1–10_) [46, 47]. Wherever the two organelles are sufficiently close to one another, bimolecular complementation of GFP [47, 48] occurs, resulting in the generation of a fluorescent mark that can be detected at the contact points. First, different combinations of split GFP were tested to verify correct expression and complementation. HeLa cells were transfected with 1 µg of the OMM plasmid GFP_β1–10_, which expresses the β1–10 portion of split GFP fused to an outer mitochondrial membrane tag, in combination with an equal amount of the following plasmids: (1) Cytosolic-targeted GFP_β11_ linked to RFP as a transfection control; (2) ER GFP_β11_ short, in which β11 is linked to an ER membrane targeting tag by a short linker; and (3) ER GFP_β11_ long, in which a long linker is used to attach β11 to the ER-targeting peptide [47]. The results are shown in Fig. S1. In all cases, bimolecular expression and complementation were achieved, and the corresponding GFP signal was observed. Complementation between OMM-GFP_β1–10_ and cytosolic-GFP_β11_-RFP generated signal localized to the mitochondrial population despite the RFP signal from the GFP_β11_-RFP fusion localizing throughout the cytoplasm, verifying that GFP fluorescence is only reconstituted when both plasmids are in sufficiently close proximity. The combination of OMM-GFP_β1–10_ transfected with either the long or short ER-GFP_β11_ construct generated a fluorescent signal associated with a subpopulation of mitochondria in the perinuclear region of the cell, a part of the cell where ER−mitochondrial contacts are known to be predominantly located under resting conditions.

**Fig. 2:**
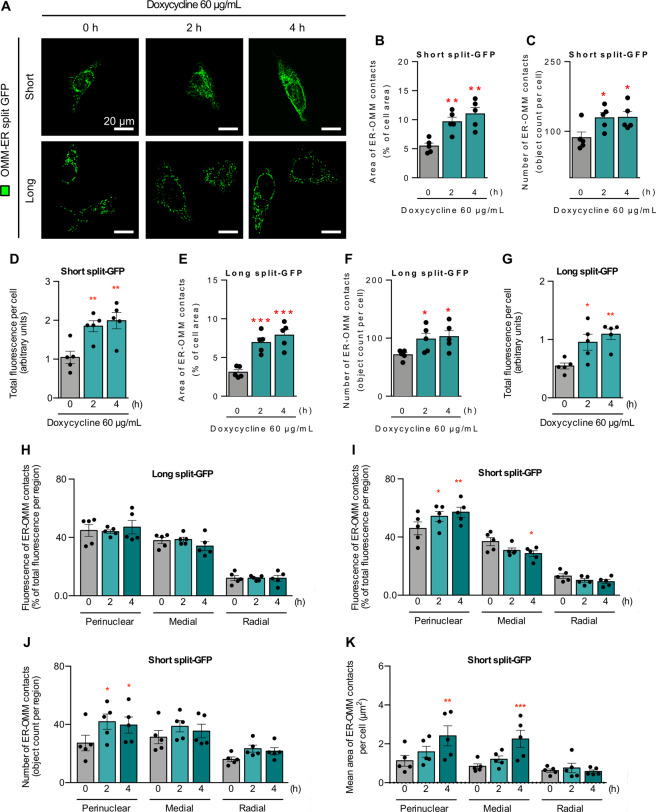
Doxycycline increases early ER−mitochondria contacts. **A** HeLa cells were transfected with two ER-mitochondria split-GFP combinations of plasmids (OMM GFPβ1–10 + [ER GFPβ11 short or ER GFPβ11 long]) for 24 h, then treated with 60 µg/mL doxycycline for the indicated times and imaged using confocal microscopy. Scale bars: 20 µm. Quantification of split-GFP fluorescence measured as the percentage of fluorescent area versus total cell area (**B**), the number of fluorescent objects (**C**) for short ER-mitochondria split-GFP, and (**D**) the total GFP fluorescence intensity per cell (*n* = 5). Quantification of split-GFP fluorescence measured as the percentage of fluorescent area versus total cell area (**E**), the number of fluorescent objects (**F**) for long ER-mitochondria split-GFP, and (**G**) the total GFP fluorescence intensity per cell (*n* = 5). **H, I** Quantification of the total GFP fluorescence intensity per cell in the predefined subcellular regions for both long and short split-GFPs (*n* = 5). Quantification of short split-GFP fluorescence measured as the number of fluorescent objects (**J**) and their size (**K**) in the predefined subcellular regions (*n* = 5). Results are shown as mean ± s.e.m. *P < 0.05, **P < 0.01, and ***P < 0.001 and versus control condition.

After validating the expression and complementation of the various plasmid combinations, doxycycline was added to the media at a final concentration of 60 µg/mL, then assessed 2 and 4 h later. Figure 2A shows representative images for each time point. Doxycycline treatment increased the fluorescence signal from OMM-GFP_β1–10_ combined with either the long or the short linker ER-GFP_β11_ construct. Three different approaches were taken to quantify the split GFP signal marking ER−mitochondrial contacts: the total fluorescence intensity per cell, the percentage of the cell area occupied by fluorescence, and the number of fluorescent objects per cell [46, 47]. Quantification indicated enhanced ER−mitochondrial contacts assessed as increases in the intensity, the number of contact points between ER and mitochondria, and the extent of the area covered by these contacts (Fig. 2B−G). For the long split-GFP, the radial distribution of the contacts remained unchanged, indicating a uniform disposition throughout the cell, similar to the results with indirect immunofluorescence. The short split-GFP, however, showed an enrichment in the perinuclear and medial regions (Fig. 2H, I). This suggests that tighter contacts form in these regions compared to the radial region. The fact that inmmunofluorescence cannot discriminate different levels of proximity agrees with its lower resolution (~200 nm). Further analysis of the tighter contacts shows that their number increases in the perinuclear region, observed as early 2 h. Meanwhile, the size of these contacts showed significant increases only at 4 h, in both the perinuclear and medial regions (Fig. 2J, K). Taken together, these results suggest that the augmentation in doxycycline-induced ER−mitochondria contacts encompasses an overall increase in ER−mitochondria proximity, characterized by an early nucleation of narrow contacts in the perinuclear region, and the subsequent growth in the size of said contacts.

### Doxycycline increases the capacity for ER-mitochondrial Ca^2+^ transfer

**Fig. 3:**
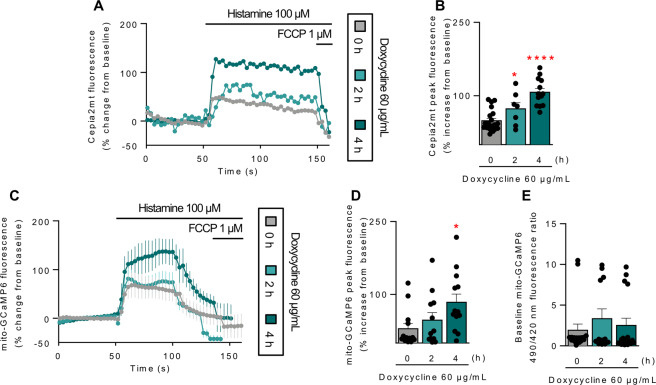
Doxycycline increases ER-to-mitochondria calcium transfer. **A** HeLa cells were transfected with the mitochondrial Ca^2+^ sensor Cepia2mt for 24 h, then treated with 60 µg/mL doxycycline for the indicated times and imaged using confocal microscopy. Baseline fluorescence was evaluated, and then Ca^2+^ release from ER stores was induced with 100 nM histamine. **B** Quantification of Cepia2mt peak fluorescence of graphs obtained in panel (**A**). For each independent imaging experiment, 7−20 cells were analyzed. **C** HeLa cells were transfected with the ratiometric mitochondrial Ca^2+^ sensor mito-GCaMP6 for 24 h, then treated with 60 µg/mL doxycycline for the indicated times and imaged using confocal microscopy. Baseline fluorescence was evaluated, and then Ca^2+^ release from ER stores was induced with 100 nM histamine. **D** Quantification of mito-GCaMP6 peak fluorescence of graphs obtained in panel (**C**). **E** Quantification of mito-GCaMP6 basal fluorescence of graphs obtained in panel (**C**). For each independent imaging experiment, 12–17 cells were analyzed. Results are shown as mean ± s.e.m. *P < 0.05 and ***P < 0.001 versus control condition.

To test whether the doxycycline-induced increases in the physical coupling between ER and mitochondria translated into an increase in functional, inter-organellar communication, we assessed the capacity for Ca^2+^ transfer from the ER to the mitochondria as this is one of the best indicators of the functionality of these contacts [49–51]. HeLa cells were transfected with Cepia2mt, a Ca^2+^ sensitive fluorescent protein targeted to the mitochondrial matrix [52]. Forty-eight hours later, the cells were treated with doxycycline or vehicle. Histamine was used to induce Ca^2+^ release from the ER at baseline as well as 2 and 4 h after doxycycline treatment. Changes in the intensity of the fluorescent signal from the Cepia2mt sensor were analyzed in real-time using confocal microscopy. Figure 3A shows that in control cells, histamine produced a rapid rise in fluorescence intensity, indicating the entry of Ca^2+^ into mitochondria. This was followed by a slow decay of the signal. After 2 h of doxycycline treatment, there was a small but significant increase in histamine-induced Ca^2+^ uptake. By 4 h post-doxycycline, there was a robust increase in the Cepia2mt fluorescence response compared to control. Furthermore, the signal remained significantly elevated compared to controls throughout the time course of the assay (Fig. 3A, B). The Cepia2mt sensor can only quantify changes in the relative levels of mitochondrial Ca^2+^. It does not allow direct comparison of differences in the basal concentration of mitochondrial Ca^2+^ or account for the differences in sensor expression from experiment to experiment under different conditions. For this reason, mitochondrial Ca^2+^ uptake experiments were repeated using a second mitochondrial-targeted, ratiometric Ca^2+^ sensor, mito-GCaMP6m, which has a fluorescence emission line that is independent of Ca^2+^, to which the Ca^2+^-dependent signal can be normalized [53]. As seen in Fig. 3C, the mito-GCaMP6m sensor yielded results similar to those obtained with the Cepia2mt sensor. Doxycycline treatment increased the transfer of Ca^2+^ from the ER to the mitochondria, with maximum fluorescence (mitochondrial Ca^2+^ uptake) greater after 4 h of treatment than after 2 h (Fig. 3D). Comparison of the basal concentration of mitochondrial Ca^2+^, before histamine stimulation, indicated that there was no significant difference in the resting levels of mitochondrial Ca^2+^ in doxycycline-treated cells compared to in control cells (Fig. 3E). Together, these data indicate that doxycycline treatment increases ER-to-mitochondria communication.

### Doxycycline treatment increases mitochondrial respiration

**Fig. 4:**
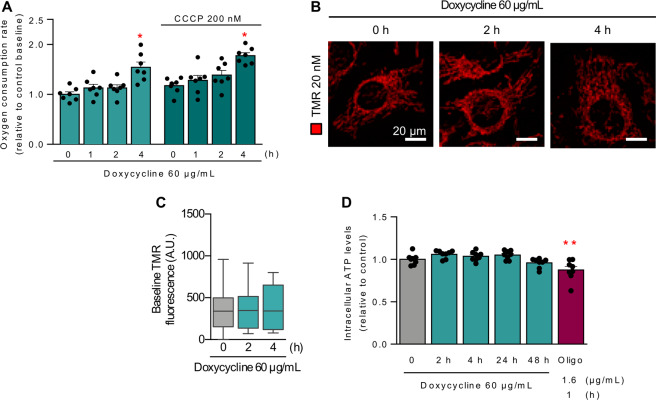
Doxycycline increases early respiration. **A** HeLa cells were treated with 60 µg/mL doxycycline for the indicated times, and then mitochondrial respiration rates were measured using a Clark electrode. CCCP 200 nM was used to analyze maximum respiration (*n* = 7). **B** HeLa cells were treated with 60 µg/mL doxycycline for the indicated times, then incubated with TMRM 20 nM for 30 min and analyzed by confocal microscopy. **C** Quantification of basal fluorescence of images obtained in panel (**B**) (*n* = 5). **D** HeLa cells were treated with 60 µg/mL doxycycline for the indicated times, and the relative levels of intracellular ATP were measured by a luminescence-based kit. Oligomycin 2 µM for 1 h was used as a technique control (*n* = 8). Results are shown as mean ± s.e.m. Boxplots are presented in Tukey’s format. *P < 0.05 and **P < 0.01 and versus control condition.

We next sought to assess whether the increase in ER−mitochondria coupling and mitochondrial Ca^2+^ uptake translated into changes in cellular metabolism. Previous studies from our group demonstrated that the response to mild ER stress improved adaptation to adverse conditions by increasing ER−mitochondria contacts and the capacity for mitochondrial Ca^2+^ uptake, thereby increasing the rate of oxygen consumption and cellular ATP levels [7, 8]. To evaluate whether mild mitochondrial stress leads to similar metabolic adaptation, we assayed the rate of oxygen consumption as a measurement of mitochondrial metabolism. There was a significant increase both in basal and uncoupled respiration after 4 h of doxycycline treatment (Fig. 4A), but not after only 2 h, indicating that an increase in ER−mitochondrial coupling precedes the increase in metabolic activity. Interestingly, increased respiration at 4 h was not accompanied by significant changes in mitochondrial membrane potential (Fig. 4B, C) or cellular ATP levels (Fig. 4D), suggesting that the ATP being generated may be being consumed by increased cellular demand. Taken together, these results indicate that an early response to mild mitochondrial stress includes an increase in oxidative metabolism. We speculate that this increase may help to maintain the levels of ATP under this stressful condition.

### Doxycycline activates a transcriptional mitochondrial stress response

**Fig. 5:**
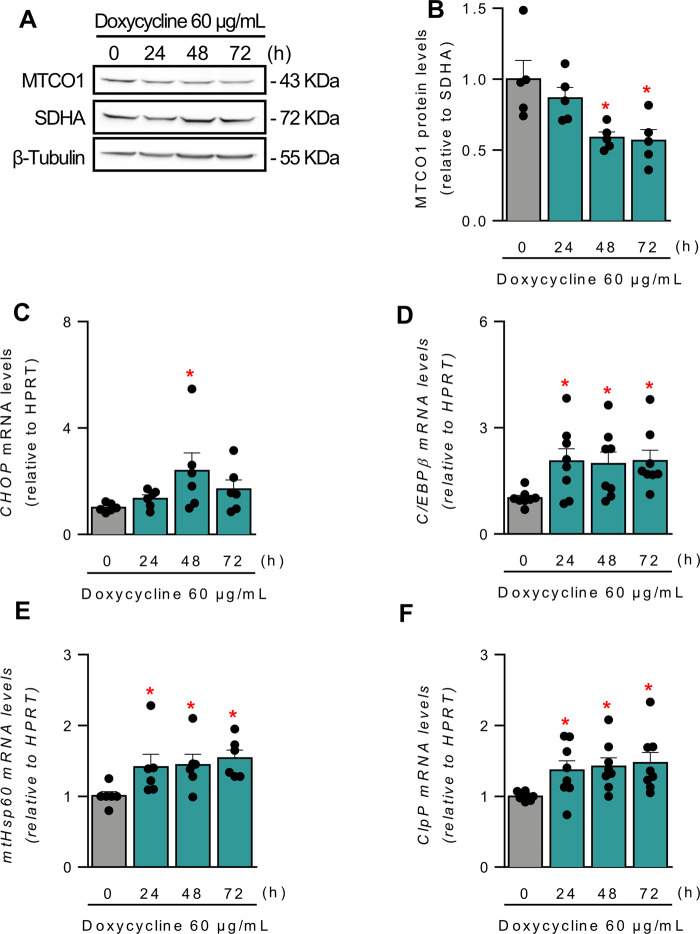
Doxycycline induces mito-nuclear protein imbalance and transcriptional mitochondrial stress response. **A**, **B** HeLa cells were treated with 60 µg/mL doxycycline for the indicated times, and MTCO1 (mitochondrial DNA-encoded) and SDHA (nuclear DNA-encoded) levels were analyzed by western blotting using β-tubulin as a loading control. **A** Representative blot. **B** Densitometric quantification (*n* = 5). **C**−**F** HeLa cells were treated with 60 µg/mL doxycycline for the indicated times, and the relative mRNA levels of *chop* (**C**)*, c/ebpβ* (**D**)*, mtHsp60* (**E**), and *clpp* (**F**), as markers for mtUPR, were analyzed by RT-qPCR, using *hprt* as a reference gene (*n* = 5–8). Results are shown as mean ± s.e.m. *P < 0.05 versus control condition.

To better understand the nature of the stress imposed by doxycycline treatment, we first wanted to confirm the effect of doxycycline on mitochondrial protein expression. As described [33, 45], doxycycline exposure induced an imbalance in the protein levels of a mitochondrial DNA-encoded subunit of the ETC (MTCO1) versus a nuclear DNA-encoded subunit (SDHA), evaluated by Western blot in total cell extracts (Fig. 5A, B). To assess whether the doxycycline treatment leads to a mitochondrial protein imbalance sufficient to induce mtUPR signaling, transcript levels for marker genes indicative of a mtUPR response were quantified using RT-qPCR. As seen in Fig. 5C−F, there was a significant increase in the transcript levels of CHOP, C/EBPβ, ClpP, and mtHsp60, consistent with that reported in the literature [33]. Of note, we also evaluate the doxycycline effect on the expression of *Xbp1* and *Hspa5*, classical targets genes activated during an ER UPR. Transcript levels were not elevated for either of these genes (Fig. S2), indicating that the response to doxycycline is specific to mtUPR signaling and not causing activation of ER UPR signaling.

### Doxycycline does not induce mitophagy nor apoptosis

**Fig. 6:**
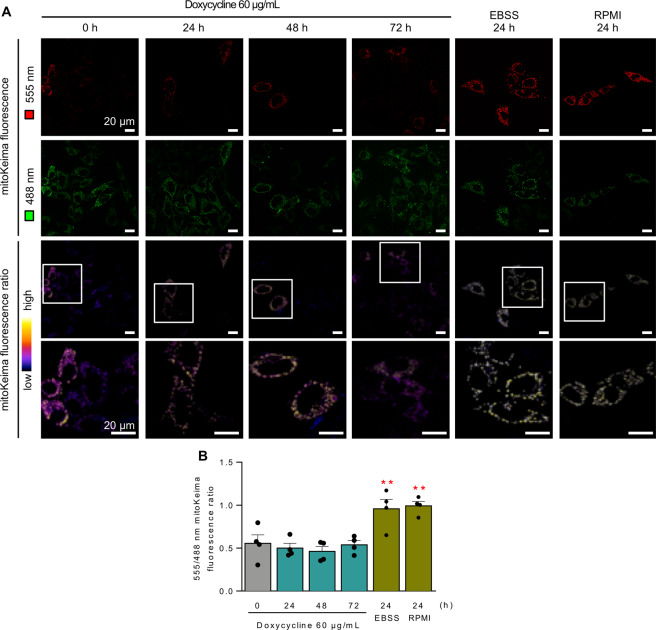
Doxycycline does not induce mitophagy. **A** HeLa cells were treated with 60 µg/mL doxycycline for the indicated times. Forty-eight hours before the end of the stimuli, the cells were transfected with mitoKeima plasmid. Representative confocal microscopy images for each condition, after exciting at 488 and 555 nM. The bottom panels show the 555/488 nm fluorescence ratio in pseudo color. EBSS (amino acid deprivation) and RPMI (glucose deprivation) for 24 h were used as positive controls for mitophagy. Scale bars: 20 µm. **B** Quantification of 555/488 nm mitoKeima emission ratio (*n* = 4). Results are shown as mean ± s.e.m. **P < 0.01 versus control condition.

Next, in order to evaluate whether the mitochondrial stress caused by doxycycline increases mitophagic activity, we transfected cells with a plasmid encoding for mitochondria-targeted Keima (mitoKeima) [54–56], a mitochondrial-targeted fluorescent protein whose emission spectra varies with pH, thereby indicating the fusion of mitochondria-containing autophagosomes with lysosomes, during the process of mitophagy. We first validated this genetic probe with known mitophagy inducers to have a positive control for mitophagic activity in HeLa cells. Either glucose deprivation (RPMI medium) or amino acid starvation (EBSS medium) were potent inducers of mitophagy (Fig. S3). Using these stimuli as positive controls for mitophagy, we evaluated the effect of late doxycycline treatment on mitoKeima fluorescence, finding no significant difference in doxycycline-treated cells compared to control cells (Fig. 6). This result indicates that the mitochondrial stress caused by doxycycline does not generate sufficient damage to the organelle to trigger mitophagic degradation quality control mechanisms. This is also consistent with the preservation of mitochondrial membrane potential seen in Fig. 4.

**Fig. 7:**
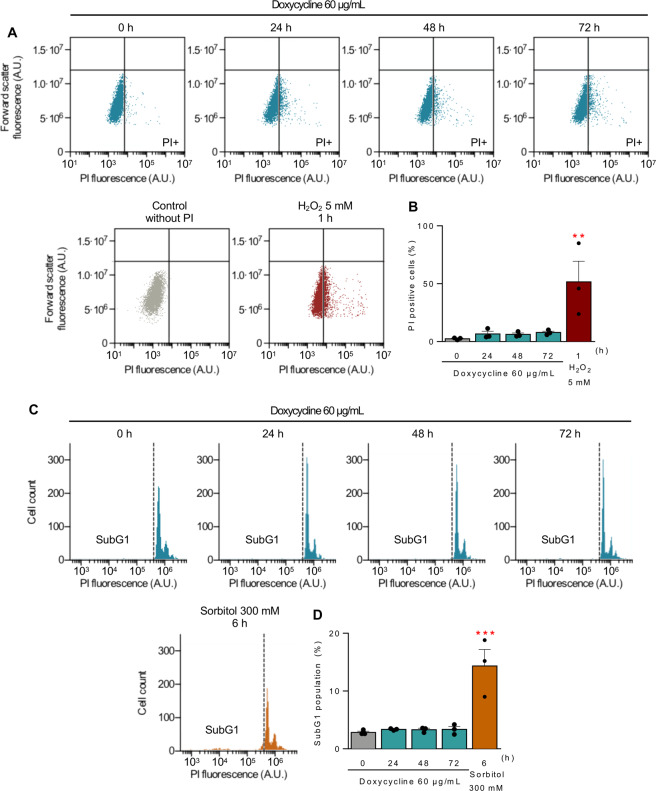
Doxycycline does not induce cell death. **A**, **B** HeLa cells were treated with 60 µg/mL doxycycline for the indicated times. Total cell death was measured as the incorporation of PI in non-permeabilized cells by flow cytometry. **A** Representative plots of relative cell size (FSC) versus PI fluorescence emission. The threshold was set according to the fluorescence emitted by a sample without PI. H_2_O_2_ 5 mM for 1 h was used as a positive control of cell death. **B** Quantification of the percentage of positive PI cells (*n* = 3). **C, D** HeLa cells treated as in panel (**A**) were fixed and permeabilized with cold methanol and stained with PI to analyze the sub-G1 population as a measure of apoptosis by flow cytometry. **C** Representative plots of cell count versus PI fluorescence emission. Sorbitol 300 mM for 6 h was used as a positive control of apoptosis. **D** Quantification of the percentage of sub-G1 cells (*n* = 3). Results are shown as mean ± s.e.m. **P < 0.01 and ***P < 0.001 versus control.

To evaluate doxycycline’s potential cellular toxicity, cell viability was assessed by flow cytometry following propidium iodide (PI) incorporation of live cells. There was no significant increase in the number of PI-positive cells after doxycycline treatment (Fig. 7A, B). Furthermore, there was no increase in the percentage of cells in the sub-G1 state, an indicator of apoptosis (Fig. 7C, D).

## Discussion

Inter-organelle communication is an essential component of cell function and adaptation, particularly under conditions of stress, including disruptions in proteostasis [1]. In this study, we asked whether stress signals originating in mitochondria would cause adaptative changes in ER−mitochondria communication. We chose to use doxycycline treatment, which is a known inductor of mitonuclear protein imbalance and concomitant mtUPR signaling [33, 40] and we showed that treatment with doxycycline increased ER−mitochondria contacts, reducing the physical distance between the two organelles. Interestingly, our data suggest that the formation of mtUPR-induced ER−mitochondria contacts is a two-step process initiated by an early nucleation and a posterior growth in size. To our knowledge, this is the first report of ER−mitochondria contacts kinetics; however, further studies are required to fully understand inter-organelle dynamics.

Our group has previously shown that an increase in ER−mitochondria contacts is an early response to ER stress, such as that induced by tunicamycin, or to a more generalized cytosolic stress, such as that induced by rapamycin [7, 8]. Importantly, unlike those, doxycycline does not cause ER stress. This study demonstrates that similar to ER stress, mitochondrial stress can promote the movement of mitochondria towards the vicinity of ER, however, the distribution of ER−mitochondrial contacts is very different under the two types of stress conditions. In a previous report, we showed that the type of stress determines the spatial distribution pattern of newly derived ER−mitochondria contacts [8]. Tunicamycin-induced ER stress led to an increase in ER−mitochondria contacts primarily in the perinuclear region of the cell, where most protein folding takes place, and which would thus be the focal point of increased demand for ATP to power the machinery required to restore protein homeostasis. In contrast, rapamycin, a mimic of nutrient starvation mimic, increased ER−mitochondria coupling throughout the entire cell [7, 8]. Here, we observed that doxycycline-induced global changes in ER−mitochondrial contacts, with a pattern similar to that observed for rapamycin, and apparently involving the movement of mitochondria toward ER rather than vice versa. However, said proximity is not uniform, because we detected tighter contacts in regions proximal to the nucleus compared to the cell periphery. Although the specifics of cellular signaling involved in the movement of mitochondria in response to doxycycline under these conditions is still unknown, we can deduce that doxycycline treatment affects the mitochondrial population throughout the entire cell, thus, the increase in ER−mitochondria contacts occurs also globally. Nevertheless, the heterogeneity in the degree of contact proximity, number, and size suggests that in different cellular regions ER−mitochondria contacts perform different processes. In vivo, it is unlikely that the entire mitochondria population in a cell is stressed uniformly. It is interesting to speculate whether regional mitochondrial stress in a subpopulation of mitochondria can lead to restricted, regional changes in ER−mitochondrial coupling.

It is widely reported that contact sites between ER and mitochondria carry out a variety of functions, including the transfer of metabolites and second messengers [16, 17, 51, 57, 58]. In this study, we evaluated the transfer of calcium from the ER to the mitochondrial matrix as a way of assessing the functionality of communication between these organelles. As previously seen, a discrete increase in mitochondrial calcium can boost mitochondrial metabolism by activating dehydrogenases in the Krebs cycle [17, 49, 57]. This could explain the observed increase in oxygen consumption after stimulation with doxycycline. However, it would be short-sited to presume that Ca^2+^ is the only molecule involved in this adaptive process. It is interesting to investigate the potential transfer and mechanism of action of other molecules that might participate in this adaptive response.

It is also relevant to note that ER−mitochondria contact sites have been shown to modulate the replication and distribution of mitochondrial nucleoids [59, 60]. As doxycycline alters mitochondrial translation, increased ER−mitochondrial contacts may be part of an adaptive response attempting to compensate for this lack of translation, augmenting mitochondrial DNA replication or distribution along with the mitochondrial network. It would be interesting to address this potential mechanism in future studies.

Beyond early-response changes in ER−mitochondria communication and mitochondrial metabolism, doxycycline did not display long-term toxicity, as cell viability remained unaffected. This agrees with a previous study, which showed that doxycycline alone does not generate apoptosis [61]. Furthermore, in our experimental setting, doxycycline did not significantly increase mitophagy, which differs from evidence showing that doxycycline stimulates mitophagy in other cell types [62]. Our model of study, HeLa cells, reportedly lack Parkin, a key protein that couples mitochondrial depolarization to mitophagy [63], which may explain their relative resistance to mitophagy. Indeed, we did not observe increased mitophagy upon CCCP exposure (a potent mitochondrial depolarizing agent), in contrast to oligomycin/antimycin treatment (which severely impair mitochondrial bioenergetics) or EBSS or RPMI, a pair of autophagy inducers (Fig. S3). Despite this, it should be noted that we resorted to short-term doxycycline treatments; however, longer use of tetracyclines, such as in inducible genetic models, can have confounding effects due to alterations in mitochondrial translation [45].

Whether the remodeling of ER−mitochondrial contacts is an early response that is an integral component of the retrograde signaling pathway remains to be determined. Although no intervention experiments were performed in this study, it could be speculated that the long-term lack of toxicity of doxycycline may be due in part to the early metabolic adaptations driven by the changes in ER−mitochondrial contacts working in conjunction with the later changes in gene expression typical of the mtUPR. It would be interesting to address these questions in future studies. mtUPR has been associated with various long-term beneficial effects in different models, so it will be important to understand better the role of early changes in communication between organelles in the long-term effects of mitochondrial stress.

## Materials and methods

### Reagents

Chemicals for general-purpose solutions were from Merck Millipore (Burlington, MA). Anti-β-tubulin antibody, DMEM (D1152), Earle´s Balanced Salt Solution (EBSS E2888), doxycycline (D9891), propidium iodide (PI, P4170), RNase (R6513), carbonyl cyanide m-chlorophenyl hydrazone (CCCP, C2759), carbonyl cyanide-4-trifluoromethoxy phenylhydrazone (FCCP, C2920), histamine (H7250), and other reagents were from Sigma-Aldrich Corp (Munich, Germany). Tunicamycin was from Enzo Life Sciences (Farmingdale, NY). Alexa fluorescent secondary antibodies, TRIzol reagent, PowerUp SYBR Green Master Mix, Opti-MEM, and Lipofectamine 3000 were from Thermo Fisher Scientific (Waltham, MA, USA). Protein assay reagents and 5x iScript RT Supermix kit were from Bio-Rad (Hercules, CA).

### Cell culture

Wild-type HeLa (CCL-2) cell line was obtained from American Type Culture Collection (ATCC, Manassas, VA). Cells were maintained in DMEM supplemented with 10% FBS, 1 mM pyruvate, and Penicillin-Streptomycin-Amphotericin B antibiotics (Biological Industries, Beit-Haemek, Israel), as described before [7, 8]. Cells were cultured in a 5% CO_2_ atmosphere at 37 °C and were used in passages 3–12.

### Transient transfection

Cells were seeded in six-well dishes at 60% confluence and transfected using OptiMEM and Lipofectamine 3000 (Thermo Fisher Scientific), according to the manufacturer’s specifications. Cells were transfected with the following plasmids: pLVX-Puro mitoKeima [54–56, 64, 65], obtained from Dr. Toren Finkel, University of Pittsburgh, USA, with authorization from Dr. Atsushi Miyawaki, RIKEN Center for Brain Science, Japan; ER-mitochondria split GFP (pcDNA3 OMM GFPβ_1–10_; pDEST ER GFP_β11_ short; pDEST ER GFP_β11_ long, pDEST Cytosolic GFP_β11_ + RFP) [46, 47], kindly donated by Dr. Gyorgy Szabadkai, University College London, UK; pCMV-CEPIA2mt [52], kindly donated by Dr. Cecilia Hidalgo, Universidad de Chile, Chile. pCMV-mito-GCaMP6m [53], kindly donated by Dr. Enrique Jaimovich, Universidad de Chile, Chile. Transfected cells were maintained for 24–48 h before further experimentation to ensure adequate protein expression.

### Experimentation

To study the adaptive response to mild mitochondrial stress, HeLa cells were treated with doxycycline at a dose of 60 µg/mL for the indicated times. To assess the long-term effects of mitochondrial stress (gene expression, mitophagy, and cell death), cells were treated for 24, 48, and 72 h. To study the early response to doxycycline, cells were stimulated for 1−4 h.

### Total protein extracts

Cells were seeded in 60-mm dishes at 60% confluence and treated according to the experiment. Cells were lysed with RIPA buffer (Tris-HCl 10 mM, pH 7.4; EDTA 5 mM; NaCl 50 mM; deoxycholic acid 1%; triton X-100 1% v/v) in the presence of proteases and phosphatases inhibitor cocktails (Roche, Basilea, Switzerland). Homogenates were centrifuged at 12000 × *g* for 10 min to eliminate cellular debris, including nuclei. Protein concentrations were measured using the Bradford method [66] according to the manufacturer’s instructions (Bio-Rad). Protein extracts were denaturated with Laemmli buffer (62.5 mM Tris-base, pH 6.8; 8% glycerol; 2.3% SDS; 0.005% bromophenol blue; 5% 2-ercaptoethanol) for 5 min at 95 °C, then stored at –20 °C.

### Western blot analysis of total protein extracts

Protein extracts were separated by SDS-PAGE (10% gels) at room temperature at 80 mV and then transferred to 0.2 µm-pore nitrocellulose membranes at 4 °C at a total of 600 mA using a Mini-PROTEAN Tetra Cell and a PowerPac Basic, both from Bio-Rad. Membranes were blocked with 5% non-fat milk 0.05% Tween 20 TBS for 1 h at room temperature, then incubated with primary antibodies overnight at 4 °C. Antibody dilutions were: anti-MTCO1 (Abcam ab90668) 1:1 000; anti-SDHA (Abcam ab137040) 1:6 000; anti-β-tubulin (Sigma-Aldrich T0198) 1:5 000. After washing in 0.05% Tween TBS, blots were incubated for 2 h with anti-mouse or anti-rabbit peroxidase-conjugated secondary antibodies (Calbiochem, San Diego, CA, USA) at dilution of 1:5 000. Protein bands were detected using EZ-ECL reagents (Biological Industries) and scanned with Dyversity 4 (Syngene, India). UN-SCAN-IT (Silk Scientific, Inc., USA) was used for densitometry analysis.

### RNA extraction

Cells were seeded in 60-mm dishes at 60% confluence and treated according to the experiment. Total RNA was isolated using TRIzol reagent (Thermo Fisher Scientific) according to the manufacturer’s instructions. RNA yield was quantified using NanoDrop 2000 (Thermo Fisher Scientific).

### RT-qPCR

The retrotranscription reaction was performed using 1 µg of total RNA and the 5x iScript RT Supermix kit (BioRad) according to the manufacturer’s instructions in a Gene Cycler thermocycler (BioRad). Real-time PCR was performed with PowerUp SYBR Green Master Mix (Applied Biosystems, Foster City, CA, USA) and a StepOnePlus Real-Time PCR System (Thermo Fisher Scientific). Mitochondrial stress-related transcripts were normalized to *hprt1* mRNA. Primers sequence, concentration, and reaction efficiency are listed in Supplementary Table 1. The qPCRs for each of the biological replicates were performed in triplicate. The ratio of the expression of a given gene versus a reference gene was calculated using the Pfaffl method [67].

### Cell viability assays

HeLa cells were seeded in 12-well plates (20 × 10^3^ cells/well) and then subjected to experimental conditions. Loss of cell viability was assessed by incorporating 1 µg/mL propidium iodide (Sigma-Aldrich) in non-permeabilized cells. The percentage of cells in the subG1 phase was used as an approximation to apoptosis evaluation. To this end, cells were permeabilized overnight with cold methanol and then treated with RNase before incubation with propidium iodide. In both cases, cell fluorescence was measured by flow cytometry (BD Accuri C6).

### Mitophagy

Cells were seeded in six-well plates with 0.17-mm coverslips (80 × 10^3^ cells/well) and treated as required in each experiment. Forty-eight hours before the end of stimuli, cells were transfected with 1 µg of mitoKeima plasmid [55, 56, 64, 65], following the protocol mentioned in the Transient transfection section. At the end of treatment, cells were incubated with Krebs medium (10 mM HEPES, pH 7.4; 140 mM NaCl; 5 mM KCl; 3.5 mM CaCl_2_; 2 mM MgCl_2_; 5.6 mM glucose), and the fluorescence was registered by confocal microscopy (LSM 700, Carl Zeiss) after exciting at 488 and 555 nm.

### Immunofluorescence studies and colocalization analysis

Cells were seeded in 12-well plates with 0.17-mm coverslips (40 × 10^3^ cells/well) and treated as indicated in each experiment. Cells were then fixed with 4% paraformaldehyde, permeabilized with 0.1% Triton X-100 and blocked with 2% BSA, all in PBS. Samples were incubated with primary antibodies in 2% BSA overnight at 4 °C. Antibody dilutions were: anti-Calnexin (ER marker, Abcam ab75801) 1:200 and anti-mtHsp70 (mitochondrial marker, Thermo Fisher Scientific MA3–028) 1:500. Following incubation for 2 h with anti-mouse Alexa 568 or anti-rabbit Alex 488-conjugated secondary antibodies (dilution 1:600), coverslips were mounted on glass slides using mounting medium ProLong with DAPI (Life technologies). For the colocalization analysis, only one focal plane was analyzed in a confocal microscope (LSM 700, Carl Zeiss). Images obtained were deconvolved, and colocalization between proteins was quantified using the Manders’ algorithm and the Image J software (NIH, USA), as previously described [8, 9].

### Radial analysis

Radial analysis of fluorescence was performed as previously described [7, 8]. Each cell was analyzed individually according to their sizes, as previously published [7, 8]. In brief, a radial distance (*r*) was calculated from the cell area using the equation *A* = π*r*^2^. This corresponds to the cell radius, assuming cells were approximately circular. The center of the nucleus was used as a starting point, and three concentric rings were drawn, defining four regions: nuclear, perinuclear, medial, and radial regions. The nuclear region was excluded from the analysis, as it was irrelevant for this study. In the remaining three areas, fluorescence colocalization was analyzed, according to the experiment.

### Bimolecular complementation of GFP

Cells were seeded in six-well plates with 0.17-mm coverslips (180 × 10^3^ cells/well) and treated as required in each experiment. Twenty-four hours before the end of stimuli, cells were transfected with 1 µg of ER-mitochondria Split-GFP plasmids combination (1 µg of OMM GFP_β1–10_ + 1 µg of ER GFP_β11_ short, 1 µg of OMM GFP_β1–10_ + 1 µg of ER GFP_β11_ long, or 1 µg of OMM GFP_β1–10_ + 1 µg of Cytosolic GFP_β11_ + RFP) [46, 47], following the protocol mentioned in transient transfection section. At the end of treatment, cells were incubated with Krebs medium (10 mM HEPES, pH 7.4; 140 mM NaCl; 5 mM KCl; 3.5 mM CaCl_2_; 2 mM MgCl_2_; 5.6 mM glucose), and the fluorescence was recorded by confocal microscopy (LSM 700, Carl Zeiss) after exciting at 488 nm. Images obtained were deconvolved. The extent of ER−mitochondria contacts was quantified as the fluorescence intensity, the number of fluorescent objects, and the area of the fluorescent stain relative to the cell using the Image J software (NIH, USA).

### Assessment of mitochondrial Ca^2+^ uptake

Cells were seeded in six-well plates with 0.17-mm coverslips (180 × 10^3^ cells/well) and treated as required in each experiment. Twenty-four hours before the end of stimuli, cells were transfected with 1 µg of Cepia2mt [52] or mito-GCaMP6m [53] plasmids, following the protocol mentioned in the transient transfection section. At the end of treatment, cells were washed and incubated with Krebs medium (10 mM HEPES, pH 7.4; 140 mM NaCl; 5 mM KCl; 3.5 mM CaCl_2_; 2 mM MgCl_2_; 5.6 mM glucose). To study histamine-induced mitochondrial calcium uptake, cells were transfected with Cepia2mt and observed by confocal microscopy (LSM 700, Carl Zeiss) with a Plan-Apochromat 63 × /1.4 Oil DIC objective, after exciting at 488 nm. Images were acquired at 1 s intervals. Basal fluorescence was measured for 50 s, and then histamine 100 µM was added, and signals were imaged for 100 s. Finally, FCCP 1 µM was added as a control of the experiment. Data are expressed as fluorescence change relative to basal values ([F–F0]/F0). To analyze basal mitochondrial calcium levels, cells were transfected with the ratiometric probe mito-GCaMP6m, and fluorescence was recorded in a spinning disk microscope (Model IX81, Olympus), after exciting at 490 nm (sensitive to Ca^2+^) and 420 nm (insensitive to Ca^2+^). Basal fluorescence was measured for 50 s, and 100 µM histamine was added, and signals were imaged for 100 s. Finally, FCCP 1 µM was added as a control of the experiment.

### ATP measurement

Cells were plated in 96-well plates (20 × 10^3^ cells/well), and ATP content after doxycycline treatment was determined using a luciferin/luciferase assay (Cell-Titer Glo Kit; Promega, Madison, WI), following the manufacturer’s instructions. Luminescence was measured using a Glomax Multidetection System (Promega). Oligomycin 2 µM for 1 h was used as a control of the technique.

### Mitochondrial potential

Cells were seeded in six-well plates with 0.17-mm coverslips (200 × 10^3^ cells/well) and treated as required in each experiment. Thirty minutes before the end of treatment, the probe tetramethylrhodamine methyl ester (TMRM) 20 nM was added to the medium. Then, coverslips were washed and maintained in Krebs solution (10 mM HEPES, pH 7.4; 140 mM NaCl; 5 mM KCl; 3.5 mM CaCl_2_; 2 mM MgCl_2_; 5.6 mM glucose) with 20 nM TMRM. Basal real-time fluorescence was recorded by confocal microscopy (LSM 700, Carl Zeiss) after exciting at 555 nm.

### Oxygraphy

Cells were seeded in 60-mm dishes at 80% confluence and treated according to the experiment. Cells were trypsinized, and the resulting suspension was placed in a chamber with a Clark electrode (Clark Oxygraph Plus System, Hansatech, King’S Lynn, Norfolk, UK), which measures oxygen consumption in living cells. After measuring basal respiration for 3 min at 25 °C, CCCP 200 nM was added to measure uncoupled respiration for another 3 min. Finally, the cell suspension was recovered to quantified total proteins using the Bradford method [66] to normalize oxygen consumption.

### Statistical analysis

Data are shown as mean ± s.e.m. of the number of independent experiments indicated (*n*). Data were analyzed using one-way ANOVA, because of the normality assumption and the number of groups. For radial analysis, two-way ANOVA was used, considering the radial region as a stratifying variable. Comparisons between groups were performed using a Holm−Sidak post-test. In all experiments, variances between groups were not statistically different, as indicated by both Bartlett’s and Brown−Forsythe’s tests. Statistical significance was determined using a 95% confidence level (*P* < 0.05). For each experiment, the sample size was determined based on our previous studies [7–9]. Investigators were not blinded during experimentation. Quantifications were automated and performed in parallel using the same parameters.

## Supplementary information

Supplementary figure 1

Supplementary figure 2

Supplementary figure 3

Supplementary table 1

## Data Availability

The datasets used and/or analyzed during the current study are available from the corresponding author on reasonable request.
