# Supplementary figures and images for: Endoplasmic reticulum−mitochondria coupling increases during doxycycline-induced mitochondrial stress in HeLa cells
Source: Cell Death Dis. 2021 Jun 28;12(7):657. doi: 10.1038/s41419-021-03945-9 (PMC8238934; doi:10.1038/s41419-021-03945-9)

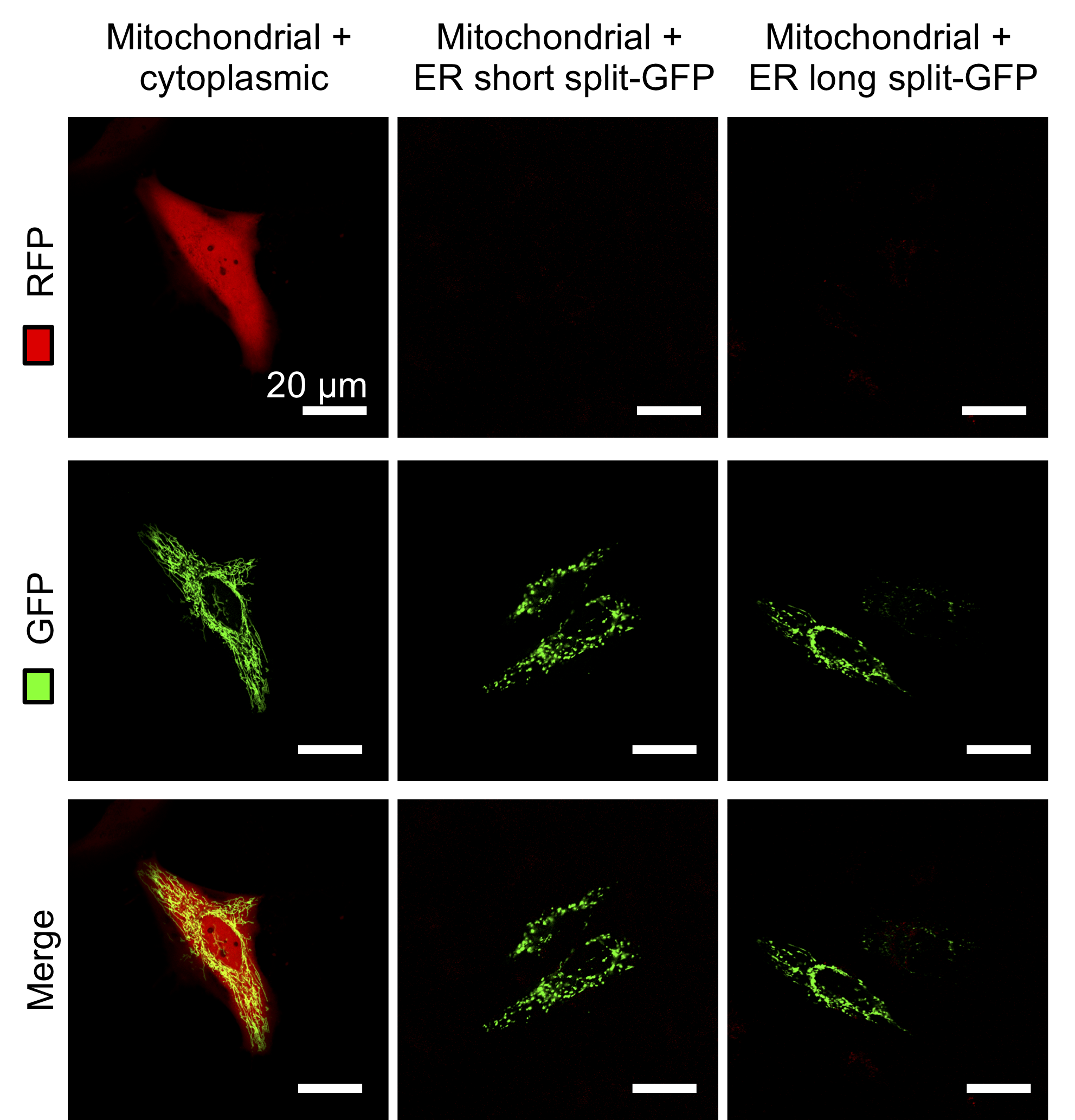

Supplement: Supplementary file 1 — Supplementary figure 1 [file 41419_2021_3945_MOESM1_ESM.png]

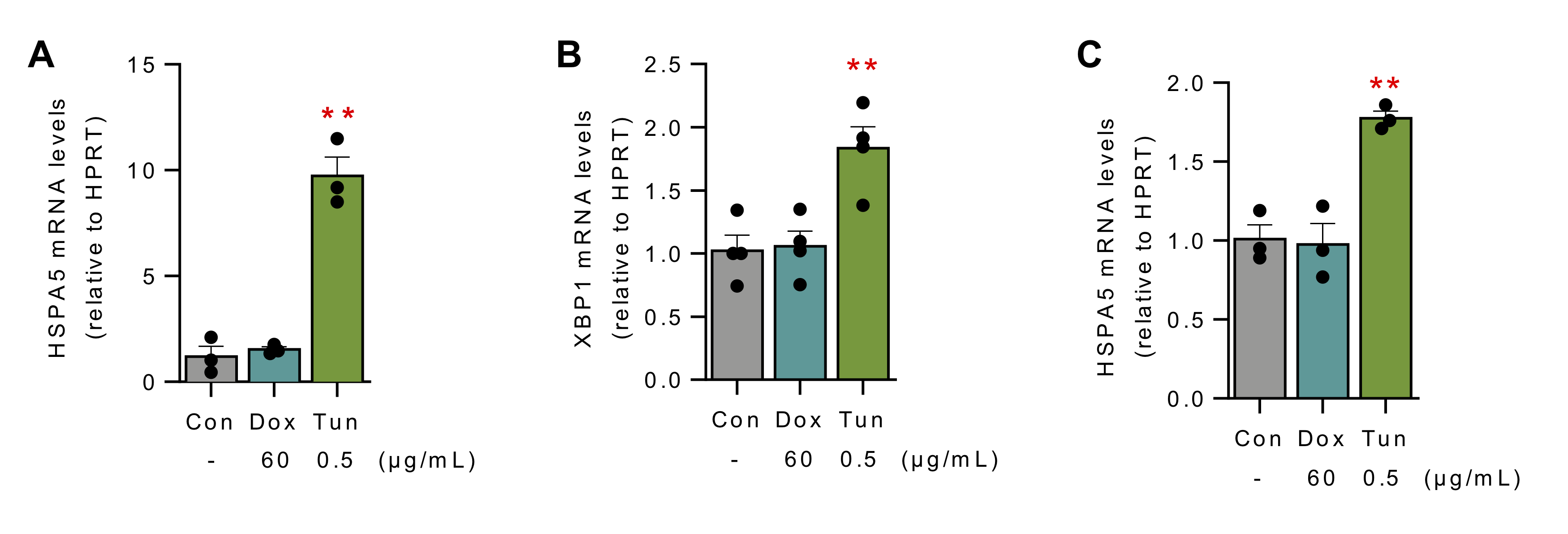

Supplement: Supplementary file 2 — Supplementary figure 2 [file 41419_2021_3945_MOESM2_ESM.png]

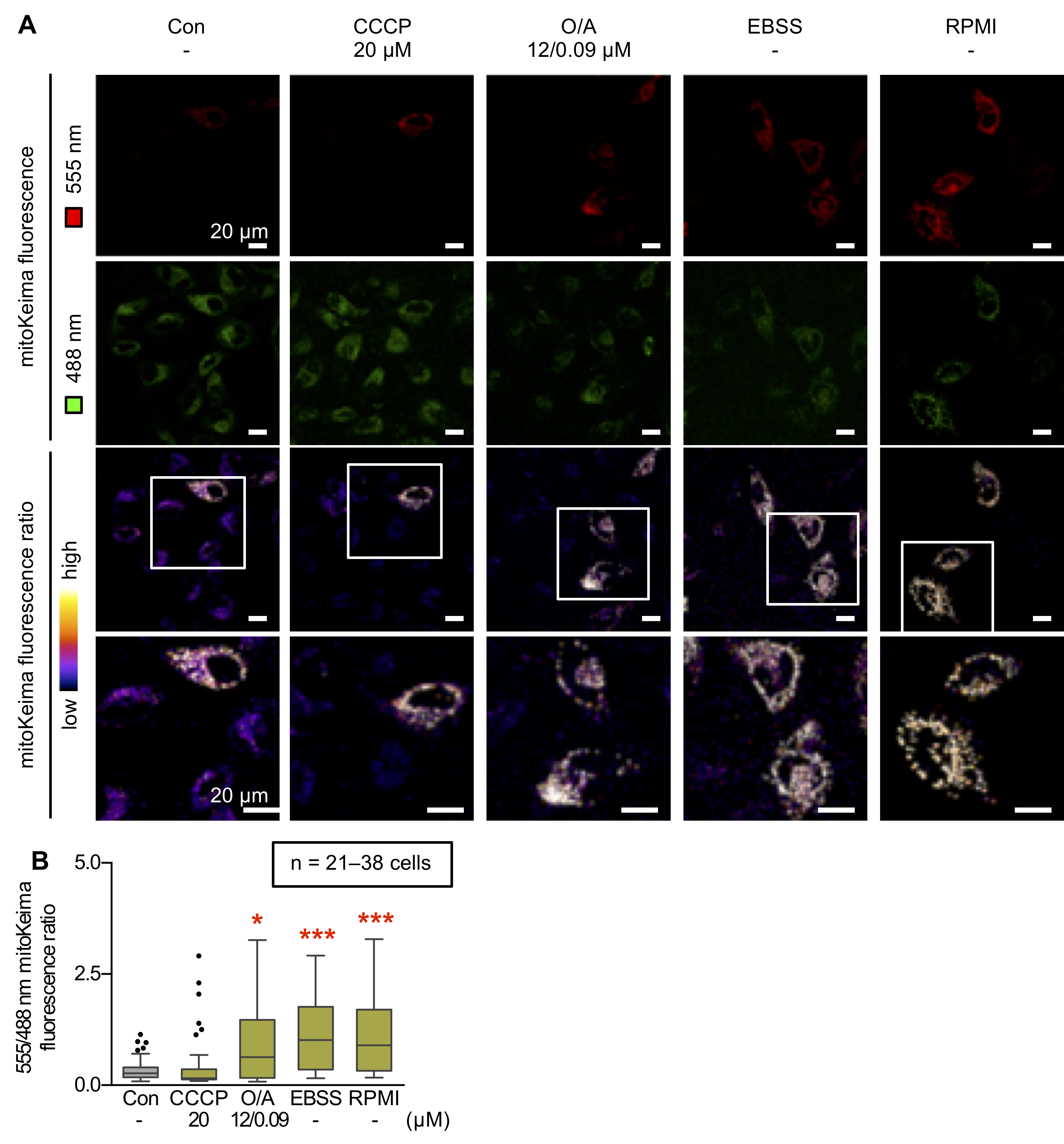

Supplement: Supplementary file 3 — Supplementary figure 3 [file 41419_2021_3945_MOESM3_ESM.png]
