# Supplementary material for: Endoplasmic reticulum−mitochondria coupling increases during doxycycline-induced mitochondrial stress in HeLa cells
Source: Cell Death Dis. 2021 Jun 28;12(7):657. doi: 10.1038/s41419-021-03945-9 (PMC8238934; doi:10.1038/s41419-021-03945-9)
Supplement: Supplementary file 4 — Supplementary table 1 [file 41419_2021_3945_MOESM4_ESM.docx]

| R^2^ | 0,966 | | 0,988 | | 0,997 | | 0,997 | | 0,963 | | 0,969 | | 0,988 | |
| --- | --- | --- | --- | --- | --- | --- | --- | --- | --- | --- | --- | --- | --- | --- |
| Efficiency (%) | 100,766 | | 98,854 | | 105,649 | | 96,339 | | 100,504 | | 94,931 | | 97,593 | |
| Concentration | 500 nM | | 500 nM | | 500 nM | | 500 nM | | 200 nM | | 200 nM | | 500 nM | |
| Sequence | 5’CCTCACTCTCCAGATTCCA 3’ | 5’AGCCGTTCATTCTCTTCAG 3’ | 5’TTACTGCTGTCATTGTCCAT 3’ | 5’GCTGCTTAACTTCTCATCTG 3’ | 5’CAGCGACGAGTACAAGAT 3’ | 5’CTGCTCCACCTTCTTCTG 3’ | 5’TGGCATCTTAGACAAGGTTC 3’ | 5’CACATGATTCTGGAGAGGAG 3’ | 5’TCTGGTGATCAAGATACAGG 3’ | 5’CTTTCACCTTCATAGACCTTG 3’ | 5’AGAGTCTGATATCCTGTTGG 3’ | 5’AGTTCATTAATGGCTTCCAG 3’ | 5’TGCTTTCCTTGGTCAGGCAGTA 3’ | 5’CAACACTTCGTGGGGTCCTTTT 3’ |
|  | Forward | Reverse | Forward | Reverse | Forward | Reverse | Forward | Reverse | Forward | Reverse | Forward | Reverse | Forward | Reverse |
| Gene | CHOP  (human) | | mtHsp60  (human) | | C/EBPβ  (human) | | ClpP  (human) | | Hspa5  (human) | | Xbp1  (human) | | Hprt  (human) | |
